# Phages against killer superbugs: An enticing strategy against antibiotics-resistant pathogens

**DOI:** 10.3389/fphar.2023.1036051

**Published:** 2023-01-24

**Authors:** Bikash Baral

**Affiliations:** Organismal and Evolutionary Biology Research Programme, Faculty of Biological and Environmental Sciences, University of Helsinki, Helsinki, Finland

**Keywords:** therapeutic leads, multi-drug resistant pathogens, biocide, antimicrobial resistance, antibiotics

## Abstract

The emerging resistivity of antibiotic resistance superbugs desire the need to resolve the global problem of antibiotic resistance. Among several other methods currently being adopted, one possible solution may be the development of supplemental therapies for antibiotics. The use of the normal and advanced bactericidal properties of bacteriophages (bacteriophage therapy) may be one of the viable infection control options. It is evident, however, that the safe and regulated application of phage treatment will need extensive knowledge of the characteristics and behaviour of certain phage–bacterium systems. This mini review offers an overview of the potential for phage therapy as well as the constraints and obstacles it faces in becoming a commonly accepted infection management strategy.

## Key messages

• Because of their ability to evolve, adapt, and target phage-resistant bacteria, phages have the potential to replace the use of conventional antibiotics in treating bacterial infections.
• Phage can be unique to species or even particular bacterium strains, making them a suitable treatment for targeting and killing infections selectively.
• Phage treatment may be preferred to antibiotic therapy for suppressing the growth of antibiotic resistant mutants with lower growth fitness, decreased pathogenicity, and defective antibiotic efflux.

## 1 Introduction

The history of research and development of antimicrobial drugs encompasses more than 15 types of antimicrobials that have become a cornerstone in the control and management of microbial infections (Aminov, 2017; Chanishvili and Aminov, 2019). In clinical medicine, antimicrobial treatment has been one of the most effective therapeutic techniques. The extensive and sometimes indiscriminate use of antibiotics in human and veterinary medicine and agriculture, however, has contributed to the widespread of antibiotics resistance in the microbiota of many ecosystems (Aminov, 2011). The increase in multidrug resistance among bacterial pathogens that may severely limit our ability to manage infectious diseases is of particular concern. Very limited options exist to treat the so-called ESKAPE group of bacteria (*Enterococcus faecium*, *Staphylococcus aureus*, *Klebsiella pneumoniae*, *Acinetobacter baumannii*, *Pseudomonas aeruginosa*, and *Enterobacter* species) (Boucher et al., 2009). The current global death toll associated with antimicrobial resistance (AMR) is estimated to be 700,000 annually (Pokharel et al., 2019; Uddin et al., 2020). Moreover, the projected death toll due to multidrug-resistant bacterial pathogens may exceed 10 million by 2050 if no immediate measures are taken (Chanishvili and Aminov, 2019).

The major question is, what went wrong with antimicrobial therapy that was originally a very successful way of treating infectious diseases? The next question is: why does antibiotic resistance develop so fast? Thus, the basic biological processes that control the ecology and evolution of microbial ecosystems need to be further studied to understand the mechanism of antibiotic resistance. The subject of antibiotics resistance has long been considered mainly from a clinical microbiology perspective (e.g., as associated exclusively with the use or overuse, or misuse of antibiotics in human medicine). This could, certainly, be one of the factors contributing to the spread of antimicrobial resistance. However, the topic has much wider ramifications and must be taken into account.

Antibiotics play a significant role in the regulatory processes in natural habitats that are involved in many functions of microbial ecosystems (Aminov, 2009). Although acting as signalling molecules in natural environments at low concentrations (Davies, 2006; Davies et al., 2006), antibiotics are primarily used in the treatment of human and animal infectious diseases due to their bacteriostatic and bactericidal properties expressed at high concentrations. Moreover, for metaphylactic reasons where entire population is infected, the treatment with antibiotics is commonly used in animal feed at subtherapeutic concentrations. The key cause of antibiotic flux into microbial environments is the widespread and haphazard use of antibiotics in clinical medicine, veterinary medicine, agriculture, and other fields (Baral and Mozafari, 2020). These are the sites where genes for antibiotic resistance that naturally arise are selected and amplified. At this point, antibiotic resistance genes are integrated into the normal microbiota and there is a reduction in the cost of fitness associated with carrying the antibiotic resistance gene. Consequently, antibiotic resistance becomes very robust for eradication even in the absence of antibiotic selective pressure (Andersson and Hughes, 2011). The reservoir of antibiotic resistance genes amplified at these hot spots is then released into other ecological habitats, along with the concomitant antibiotics. These are further distributed through substantial horizontal gene transfer mechanisms to even more distant ecosystems, including pathogens (Aminov, 2011).

The prevalent antibiotic resistance in a variety of microbiota, including human and animal pathogens, is the outcome of extensive employment of antibiotics in human and veterinary medicine and agriculture (Bhattarai et al., 2020). The current question is what can be done to limit and contain it? It is apparent that it is important to minimize the extensive use of antibiotics, which eventually results in the selection of the corresponding resistance mechanisms. Unfortunately, the patterns in the production and use of antibiotics are quite the contrary, showing a quite large rise in antibiotic use for both humans and animals (Van Boeckel et al., 2014; 2015).

During the Golden Age of antibiotics discovery, which ended more than 50 years ago, all major groups of antibiotics were found (Aminov, 2017; Baral and Mozafari, 2020; Bhattarai et al., 2020; 2021). Since then, systematic modifications of known natural compounds have been involved in significant studies and developments in antimicrobial drugs. This does not, however, overcome the rapid growth of resistance even to newer antimicrobial derivatives. There are several alternative growth methods, in addition to the modification of existing bactericidal agents. Alternatives to antimicrobials are desperately needed and one of the most promising alternatives is the use of phage therapy.

## 2 Phage therapy: Beyond antibiotic resistance

**FIGURE 1:**
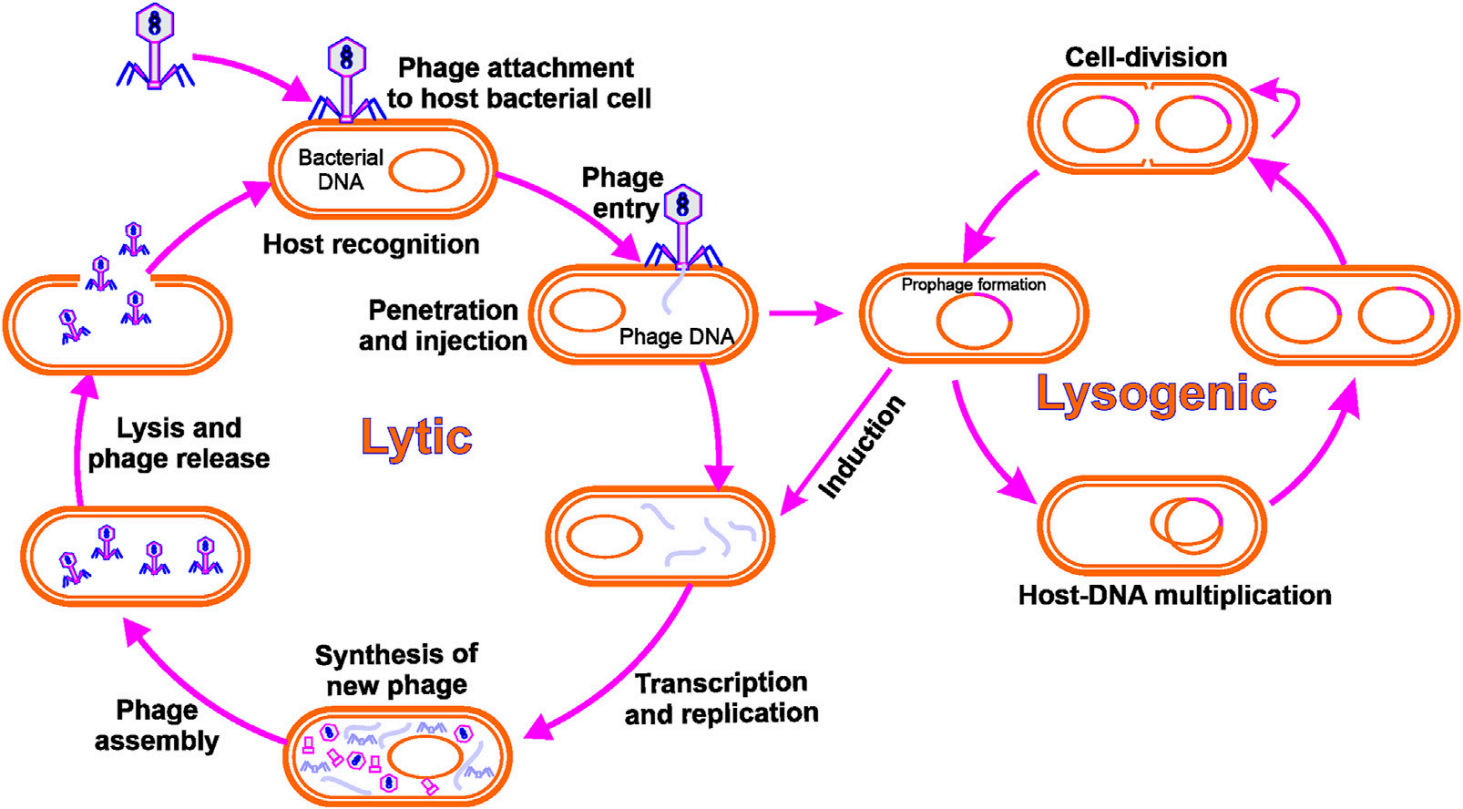
Bacteriophage life cycle: Lytic phages adhere to and infect a bacterial cell, culminating in phage reproduction and lysis of the host cell. The integration of a phage genome into the bacterial genome is the consequence of the lysogenic cycle.

Bacteriophages are specialized, bacteria-infecting viruses. They are obligatory parasites, like any other virus, requiring replication of the host cellular machinery. Infection starts with the phage particles being bound to their host cell by precise recognition of a receptor on the host surface, accompanied by phage nucleic acid delivery into the infected cell. While inside the bacterium, the phage takes over the bacterial cell, hijacking its cellular components and shutting down its defence mechanisms. Consequently, phage genes are expressed, and the genome of the phage is replicated and eventually packaged into self-assembled phage particles. Progeny phage particles emerge from the cell at the end of the lytic cycle in a process that usually includes cell lysis by the phage proteins (Calendar, 2006). To date, most isolated phages have been found to have DNA (dsDNA) linear, double-stranded genomes packed into a tailed capsid protein (Ackermann, 2007). In other groups of phages, non-tailed capsids may be present with dsDNA genomes or single-stranded DNA (ssDNA), or RNA genomes. Figure 1 demonstrates bacteriophage life cycle through different modes of replication.

The idea for the treatment of bacteriophage infections came from the groundbreaking work of D’Herelle (1917), and the first use of phages as therapeutic agents in 1919 (Hermoso et al., 2007; Ul Haq et al., 2012). However, the phage therapy approach was overshadowed by the discovery of antibiotics that provided a more convenient approach to the control of infectious diseases. It was largely abandoned worldwide, with the exception of a handful of countries: Georgia, Poland, and Russia, where it remained part of an approved therapy to treat certain bacterial infections. The need for new approaches to bacterial infection control, especially with regard to multidrug resistance, is governed by a renewed interest in phage therapy.

The use of bacteriophages in the treatment of infections provides several benefits. Firstly, phages are very particular and do not affect other beneficial microorganisms/commensal microflora, unlike the large variety of bacteria attacked by antibiotics. This avoids complications such as dysbacteriosis and secondary infections caused by antibiotics. In addition, at the locations where the targets are present, phages multiply, thus increasing local antibacterial effects. It is worth noting that no intense side effects of phage therapy have been identified so far (Borysowski and Górski, 2008; Międzybrodzki et al., 2012; McCallin et al., 2013). In particular, phage-resistant bacteria remain susceptible to other phages, and the introduction of new phages is a much faster and cheaper process, in line with Russian regulations and requirements for the production of commercial phage preparations, compared to the creation of new antimicrobials. The other advantage is that phages could be a valuable source of enzymes that are active against pathogens, such as lysine (Schmelcher et al., 2012). Bacteriophages can also play an important role in limiting the growth and spread of antibiotic resistance (Jalasvuori et al., 2011; Zhang and Buckling, 2012). It is equally important for phages to be used effectively for diagnostic purposes (O’Sullivan et al., 2016). Phages, unlike antibiotics, are also effective against biofilm-forming pathogens (Chanishvili and Aminov, 2019), and eventually, phages can supplement antibiotics, i.e., phage-antibiotic combination infection therapy is more successful than either alone (Torres-Barceló and Hochberg, 2016). Recent regulatory approval of bacteriophages for the control of foodborne pathogens as food additives has opened new possibilities for their usage in biocontrol processes (Kazi and Annapure, 2016). In addition, phage infections can target specific genes or pathways associated with biofilm formation, potentially interfering with bacteria’s capability to create biofilms (Roy et al., 2018). Moreover, some phages specifically target genes involved in exopolysaccharide production, reducing the ability of bacteria to form a protective matrix (Sharma et al., 2016).

While phages have been used for a century for the treatment of different diseases, their use in practical medicine is still restricted to several countries. When considering the use of bacteriophages for therapy and prophylaxis, protection is one of the key concerns. This is because they are living organisms, unlike typical pharmacological products. In addition, in the form of transduction, they can contribute to horizontal gene transfer (Lerminiaux and Cameron, 2019). In accordance with the technical criteria applicable in countries currently developing commercial phage preparations, several tests confirming its lytic existence and ruling out a possible involvement in horizontal gene transfer must be passed before a new phage is considered for practical use. The creation of Omics technology helps to solve this issue. In order to consider phage candidates, genome sequencing is an important initial step as it recognizes prophage genes such as integrases, repressors, excisases, recombinases, terminases and thus enables predictions of potential prophage properties, including virulence factors or incompleteness of prophage (Sybesma et al., 2018).

Regulatory problems are a big impediment to the application of phage therapy. It is important to confirm the effectiveness of phage therapy according to existing pharmacological guidelines. This includes the introduction of clinical trials that are correctly planned, randomised, placebo-controlled and double-blind. Between 1996 and 2018, 17 clinical trials were reported to this stage. Most of them could not, however, attract sufficient patients and the others were not well planned. Therefore, they are far from making scientifically valid conclusions on the effectiveness of phage therapy (Sybesma et al., 2018). For instance, a total of 27 patients from 11 centres alone were enrolled in the recently concluded Phagoburn study, which included a public expenditure of EUR 3.85 million (Jault et al., 2019). This is far lower than the 220 pre-calculated patients expected to provide statistically relevant results for the study. The trial was also designed to treat infections caused by burn wounds caused by *E. coli*, although the clinical results suggested that *P. aeruginosa* was predominant. The initial flaw in the design of the trial was greatly influenced by the insufficient test result since it is well known that burn wounds are often contaminated with *P. aeruginosa*, not *E. coli*. Georgia recently completed a randomized, placebo-controlled, double-blind clinical trial. Patients with infections of the urinary tract triggered by *E. coli*, *Enterococcus* spp., *P. aeruginosa*, *Proteus* spp., *Streptococccus* spp., *P. aeruginosa*, and *S. aureus* were enrolled in the study. Patients have been treated with a commercial Pyo-bacteriophage preparation, a mixture of bacteriophages that targets both of these pathogens. Preliminary findings have been released for the clinical trial (Leitner et al., 2017; Ujmajuridze et al., 2018), which will ideally lead to a wider recognition of phage therapy for the treatment of multidrug-resistant infections. Phage therapies will, once generally accepted, be used as an enticing and useful alternative to minimize the conventional use of antibiotics and resolve the resistance of antibiotics to pathogens.

### 2.1 Evolutionary dynamics of bacterial resistance against phages

The evolutionary dynamics of bacterial resistance to phages refers to how bacteria adapt and evolve to protect themselves from phages. Factors that influence this process include selection pressure, genetic mechanisms of resistance, trade-offs, co-evolution, and implications for human health (Hasan and Ahn, 2022). Phages can exert significant evolutionary pressure on bacterial populations, promoting the genetic changes in bacteria that allow the bacteria to survive and reproduce, eventually causing the spread of the resistant genes. Moreover, bacteria can resist phages by altering their outer membranes, producing phage replication inhibitors, or changing the phages’ target sites. This competition among bacterial members, as well as phage-bacteria co-evolution, may lead to a ‘arms race in which both sides develop new strategies. For instance, *Pseudomonas aeruginosa* isolates obtained from a wastewater treatment facility evolved phage resistance by obtaining proteins that block phage activity, allowing bacteria to proliferate in the vicinity of phages (Withey et al., 2005). Moreover, *P. aeruginosa* formed a slime layer that prevents phage attachment and aids in the spread of resistance to other *P. aeruginosa* strains (Al-Wrafy et al., 2017). In another study, *E. coli* evolved phage resistance through the acquisition of DNA mutations that altered the structure of phage target sites, hindering phage binding and infection (Meyer et al., 2012). Thus, through several means, bacteria develop resistance against phages, and phages also develop novel strategies to infect bacteria.

The trade-offs between phages and bacteria demonstrate how sophisticated evolutionary adaptation is and how different selective pressures and characteristic expressions must be considered in order to comprehend the complexities of bacterial populations. The different trade-off between phages and bacteria can be categorized into: 1) Phage-binding receptor-mediated trade-off, 2) Superinfection exclusion-mediated trade-off, 3) CRISPR–Cas-mediated trade-off, and 4) restriction modifications system-mediated trade-off (Hasan and Ahn, 2022). Thus, understanding these evolutionary dynamics is critical for managing bacterial infections and developing new antibiotic resistance strategies (Baral and Mozafari, 2020; Bhattarai et al., 2020; Mozafari et al., 2021).

### 2.2 Existing formulations of phage therapies

Currently, several phage therapy formulations are available, including 1) purified phages isolated from their natural sources, 2) phage lysates, which are the suspensions of bacterial cells infected and lysed by phages, 3) phage cocktails (mixtures of multiple different phages that may help to overcome the development of phage resistance), and 4) phage-encapsulated nanoparticles (particles containing phages within a protective shell and designed to discharge the phages in a controlled manner) and phage-derived enzymes, which are proteins generated by phages during their replication cycle (Chan et al., 2013; Malik et al., 2017; Cui et al., 2019; Nikolich and Filippov, 2020; Rosner et al., 2021). Depending on the site of the bacterial infection, these phage formulations can be administered to patients intravenously, orally, or topically (Chan et al., 2013; Malik et al., 2017).

### 2.3 Challenges of phage therapy

Though of huge therapeutic importance, phage therapy also suffers from severe drawbacks, which include 1) determining the precise type of bacteria causing an infection and linking it with the appropriate phage, 2) narrow host range, and not ideal for systemic diseases, 3) ensuring that the phage used in treatment does not harm the host, 4) overcoming bacteria’s ability to develop phage resistance, 5) phages’ limited availability and the specialized knowledge and expertise needed to use them, and 6) the potential for regulatory challenges given that phage therapy is not widely accepted (Caflisch et al., 2019; Nikolich and Filippov, 2020; Pires et al., 2020).

### 2.4 Commercialization of phage therapy

Despite the difficulties highlighted above, some biotechnology firms are still pursuing on the development of phage therapy on a commercial scale. For instance, BioLynceus (works on developing a platform for the large-scale production of phages), the Phage Therapy Center in Georgia (offers phage therapy on a commercial scale), AmpliPhi Biosciences (focuses on developing phage-based therapies for the treatment of bacterial infections), Locus Biosciences (develops engineered phage biotherapeutics), Intralytix (develops and manufactures phage-based products for the food and healthcare industries), Adaptive Phage Therapeutics (offers customized phage therapy for the treatment of bacterial infections), etc. These companies are working to circumvent phage therapy’s drawbacks and increase its acceptance as a treatment option for bacterial infections.

## 3 Conclusion

Finally, phage therapy may be a successful treatment for bacterial infections, but there are still several issues that need to be resolved before it can be extensively used. In order to overcome these difficulties and increase the usage of phage therapy as a treatment option, additional study regarding the potential risks and benefits of using phages as a therapeutic agent and its development are required.

## References

[B1] Ackermann H.-W. W. (2007). 5500 Phages examined in the electron microscope. Arch. Virol. 152, 227–243. 10.1007/s00705-006-0849-1 17051420

[B2] Al-Wrafy F. Brzozowska E. Górska S. Gamian A. (2017). Pathogenic factors of *Pseudomonas aeruginosa* – The role of biofilm in pathogenicity and as a target for phage therapy. Postepy Hig. Med. Dosw 71, 78–91. 10.5604/01.3001.0010.3792 28258668

[B3] Aminov R. (2017). History of antimicrobial drug discovery: Major classes and health impact. Biochem. Pharmacol. 133, 4–19. 10.1016/j.bcp.2016.10.001 27720719

[B4] Aminov R. I. (2011). Horizontal gene exchange in environmental microbiota. Front. Microbiol. 2, 158. 10.3389/fmicb.2011.00158 21845185 PMC3145257

[B5] Aminov R. I. (2009). The role of antibiotics and antibiotic resistance in nature. Environ. Microbiol. 11, 2970–2988. 10.1111/j.1462-2920.2009.01972.x 19601960

[B6] Andersson D. I. Hughes D. (2011). Persistence of antibiotic resistance in bacterial populations. FEMS Microbiol. Rev. 35, 901–911. 10.1111/j.1574-6976.2011.00289.x 21707669

[B7] Baral B. Mozafari M. R. (2020). Strategic moves of “superbugs” against available chemical scaffolds: Signaling, regulation, and challenges. ACS Pharmacol. Transl. Sci. 3, 373–400. 10.1021/acsptsci.0c00005 32566906 PMC7296549

[B8] Bhattarai K. Bastola R. Baral B. (2020). “Antibiotic drug discovery: Challenges and perspectives in the light of emerging antibiotic resistance,” in Advances in genetics (Academic Press), 229–292. 10.1016/bs.adgen.2019.12.002 32560788

[B9] Bhattarai K. Kabir M. E. Bastola R. Baral B. (2021). Fungal natural products galaxy: Biochemistry and molecular genetics toward blockbuster drugs discovery. 10.1016/bs.adgen.2020.11.006 33641747

[B10] Borysowski J. Górski A. (2008). Is phage therapy acceptable in the immunocompromised host? Int. J. Infect. Dis. 12, 466–471. 10.1016/j.ijid.2008.01.006 18400541

[B11] Boucher H. W. W. Talbot G. H. H. Bradley J. S. S. Edwards J. E. E. Gilbert D. Rice L. B. B. (2009). Bad bugs, No drugs: No ESKAPE! An update from the infectious diseases society of America. Clin. Infect. Dis. 48, 1–12. 10.1086/595011 19035777

[B12] Caflisch K. M. Suh G. A. Patel R. (2019). Biological challenges of phage therapy and proposed solutions: A literature review. Expert Rev. Anti Infect. Ther. 17, 1011–1041. 10.1080/14787210.2019.1694905 31735090 PMC6919273

[B13] Calendar R. (2006). The bacteriophages. Second. Oxford University.

[B14] Chan B. K. Abedon S. T. Loc-Carrillo C. (2013). Phage cocktails and the future of phage therapy. Future Microbiol. 8, 769–783. 10.2217/FMB.13.47/ASSET/IMAGES/LARGE/FIGURE2.JPEG 23701332

[B15] Chanishvili N. Aminov R. (2019). Bacteriophage therapy: Coping with the growing antibiotic resistance problem. Microbiol. Aust. 40, 5–7. 10.1071/MA19011

[B16] Cui Z. Guo X. Feng T. Li L. (2019). Exploring the whole standard operating procedure for phage therapy in clinical practice. J. Transl. Med. 17, 373–377. 10.1186/s12967-019-2120-z 31727099 PMC6857313

[B17] Davies J. (2006). Are antibiotics naturally antibiotics? J. Ind. Microbiol. Biotechnol. 33, 496–499. 10.1007/s10295-006-0112-5 16552582

[B18] Davies J. Spiegelman G. B. Yim G. (2006). The world of subinhibitory antibiotic concentrations. Curr. Opin. Microbiol. 9, 445–453. 10.1016/j.mib.2006.08.006 16942902

[B19] D’Herelle F. (1917). Sur un microbe invisible antagoniste des bacteries dysenteriques. Compt Rend. Acad. Sci. 373.

[B20] Hasan M. Ahn J. (2022). Evolutionary dynamics between phages and bacteria as a possible approach for designing effective phage therapies against antibiotic-resistant bacteria. Antibiotics 11, 915. 10.3390/antibiotics11070915 35884169 PMC9311878

[B21] Hermoso J. A. García J. L. García P. (2007). Taking aim on bacterial pathogens: From phage therapy to enzybiotics. Curr. Opin. Microbiol. 10, 461–472. 10.1016/J.MIB.2007.08.002 17904412

[B22] Jalasvuori M. Friman V.-P. P. Nieminen A. Bamford J. K. H. H. Buckling A. (2011). Bacteriophage selection against a plasmid-encoded sex apparatus leads to the loss of antibiotic-resistance plasmids. Biol. Lett. 7, 902–905. 10.1098/rsbl.2011.0384 21632619 PMC3210665

[B23] Jault P. Leclerc T. Jennes S. Pirnay J. P. Que Y. A. Resch G. (2019). Efficacy and tolerability of a cocktail of bacteriophages to treat burn wounds infected by *Pseudomonas aeruginosa* (PhagoBurn): A randomised, controlled, double-blind phase 1/2 trial. Lancet Infect. Dis. 19, 35–45. 10.1016/S1473-3099(18)30482-1 30292481

[B24] Kazi M. Annapure U. S. (2016). Bacteriophage biocontrol of foodborne pathogens. J. Food Sci. Technol. 53, 1355–1362. 10.1007/s13197-015-1996-8 27570260 PMC4984715

[B25] Leitner L. Sybesma W. Chanishvili N. Goderdzishvili M. Chkhotua A. Ujmajuridze A. (2017). Bacteriophages for treating urinary tract infections in patients undergoing transurethral resection of the prostate: A randomized, placebo-controlled, double-blind clinical trial. BMC Urol. 17, 90. 10.1186/s12894-017-0283-6 28950849 PMC5615798

[B26] Lerminiaux N. A. Cameron A. D. S. S. (2019). Horizontal transfer of antibiotic resistance genes in clinical environments. Can. J. Microbiol. 65, 34–44. 10.1139/cjm-2018-0275 30248271

[B27] Malik D. J. Sokolov I. J. Vinner G. K. Mancuso F. Cinquerrui S. Vladisavljevic G. T. (2017). Formulation, stabilisation and encapsulation of bacteriophage for phage therapy. Adv. Colloid Interface Sci. 249, 100–133. 10.1016/J.CIS.2017.05.014 28688779

[B28] McCallin S. Alam Sarker S. Barretto C. Sultana S. Berger B. Huq S. (2013). Safety analysis of a Russian phage cocktail: From MetaGenomic analysis to oral application in healthy human subjects. Virology 443, 187–196. 10.1016/j.virol.2013.05.022 23755967

[B29] Meyer J. R. Dobias D. T. Weitz J. S. Barrick J. E. Quick R. T. Lenski R. E. (2012). Repeatability and contingency in the evolution of a key innovation in phage lambda. Science 335, 428–432. 10.1126/science.1214449 22282803 PMC3306806

[B48] Mozafari (2021). Antimicrobial Applications of Nanoliposome Encapsulated Silver Nanoparticles: A Potential Strategy to Overcome Bacterial Resistance (See link: https://www.ingentaconnect.com/content/ben/cnano/2021/00000017/00000001/art00005).

[B30] Międzybrodzki R. Borysowski J. Weber-Dąbrowska B. Fortuna W. Letkiewicz S. Szufnarowski K. (2012). Clinical aspects of phage therapy. Elsevier. 10.1016/b978-0-12-394438-2.00003-7 22748809

[B31] Nikolich M. P. Filippov A. A. (2020). Bacteriophage therapy: Developments and directions. Antibiotics 9, 135. 10.3390/antibiotics9030135 32213955 PMC7148498

[B32] O’Sullivan L. Buttimer C. McAuliffe O. Bolton D. Coffey A. O’Sullivan L. (2016). Bacteriophage-based tools: Recent advances and novel applications. F1000Res 5, 2782. 10.12688/f1000research.9705.1 27990274 PMC5133683

[B33] Pires D. P. Costa A. R. Pinto G. Meneses L. Azeredo J. (2020). Current challenges and future opportunities of phage therapy. FEMS Microbiol. Rev. 44, 684–700. 10.1093/FEMSRE/FUAA017 32472938

[B34] Pokharel S. Raut S. Adhikari B. (2019). Tackling antimicrobial resistance in low-income and middle-income countries. BMJ Glob. Health 4, e002104. 10.1136/bmjgh-2019-002104 PMC6861125 31799007

[B35] Rosner D. Clark J. Schneider C. Gibb B. (2021). Formulations for bacteriophage therapy and the potential uses of immobilization. Pharmaceuticals 14, 359. 10.3390/PH14040359 33924739 PMC8069877

[B36] Roy R. Tiwari M. Donelli G. Tiwari V. (2018). Strategies for combating bacterial biofilms: A focus on anti-biofilm agents and their mechanisms of action. Virulence 9, 522–554. 10.1080/21505594.2017.1313372 28362216 PMC5955472

[B37] Schmelcher M. Donovan D. M. Loessner M. J. (2012). Bacteriophage endolysins as novel antimicrobials. Future Microbiol. 7, 1147–1171. 10.2217/fmb.12.97 23030422 PMC3563964

[B38] Sharma G. Sharma S. Sharma P. Chandola D. Dang S. Gupta S. (2016). *Escherichia coli* biofilm: Development and therapeutic strategies. J. Appl. Microbiol. 121, 309–319. 10.1111/JAM.13078 26811181

[B39] Sybesma W. Rohde C. Bardy P. Pirnay J.-P. P. Cooper I. Caplin J. (2018). Silk route to the acceptance and Re-implementation of bacteriophage therapy—Part II. Antibiotics 7, 35. 10.3390/antibiotics7020035 29690620 PMC6023077

[B40] Torres-Barceló C. Hochberg M. E. (2016). Evolutionary rationale for phages as complements of antibiotics. Trends Microbiol. 24, 249–256. 10.1016/j.tim.2015.12.011 26786863

[B41] Uddin M. J. Dawan J. Jeon G. Yu T. He X. Ahn J. (2020). The role of bacterial membrane vesicles in the dissemination of antibiotic resistance and as promising carriers for therapeutic agent delivery. Microorganisms 8, 670. 10.3390/microorganisms8050670 32380740 PMC7284617

[B42] Ujmajuridze A. Chanishvili N. Goderdzishvili M. Leitner L. Mehnert U. Chkhotua A. (2018). Adapted bacteriophages for treating urinary tract infections. Front. Microbiol. 9, 1832. 10.3389/fmicb.2018.01832 30131795 PMC6090023

[B43] Ul Haq I. Chaudhry W. N. Akhtar M. N. Andleeb S. Qadri I. (2012). Bacteriophages and their implications on future biotechnology: A review. Virol. J. 9, 9–8. 10.1186/1743-422X-9-9 22234269 PMC3398332

[B44] Van Boeckel T. P. Brower C. Gilbert M. Grenfell B. T. Levin S. A. Robinson T. P. (2015). Global trends in antimicrobial use in food animals. Proc. Natl. Acad. Sci. U. S. A. 112, 5649–5654. 10.1073/pnas.1503141112 25792457 PMC4426470

[B45] Van Boeckel T. P. Gandra S. Ashok A. Caudron Q. Grenfell B. T. Levin S. A. (2014). Global antibiotic consumption 2000 to 2010: An analysis of national pharmaceutical sales data. Lancet Infect. Dis. 14, 742–750. 10.1016/S1473-3099(14)70780-7 25022435

[B46] Withey S. Cartmell E. Avery L. M. Stephenson T. (2005). Bacteriophages—Potential for application in wastewater treatment processes. Sci. Total Environ. 339, 1–18. 10.1016/J.SCITOTENV.2004.09.021 15740754

[B47] Zhang Q.-G. G. Buckling A. (2012). Phages limit the evolution of bacterial antibiotic resistance in experimental microcosms. Evol. Appl. 5, 575–582. 10.1111/j.1752-4571.2011.00236.x 23028398 PMC3461140

